# Supplementary material for: The antidiabetic drug metformin aids bacteria in hijacking vitamin B12 from the environment through RcdA
Source: Commun Biol. 2023 Jan 24;6:96. doi: 10.1038/s42003-023-04475-0 (PMC9873799; doi:10.1038/s42003-023-04475-0)
Supplement: Supplementary file 2 — Supplementary Information [file 42003_2023_4475_MOESM2_ESM.pdf]

Supplementary information for

The antidiabetic drug metformin aids bacteria in hijacking vitamin B12 from the environment through RcdA

Authors: Luxia Yao[1,2,3,4, †], Yihan Wang[2,3,4, †], Shenlu Qin[2,3,4], Shihao Zhu[2,3,4], Lianfeng Wu[2,3,4, *]

Affiliations:

[1]Fudan University, Shanghai, China.

[2]Key Laboratory of Growth Regulation and Translational Research of Zhejiang Province, School of Life Sciences, Westlake University, Hangzhou, Zhejiang, China.

[3]Westlake Laboratory of Life Sciences and Biomedicine, Hangzhou, Zhejiang, China.

[4]Institute of Basic Medical Sciences, Westlake Institute for Advanced Study, Hangzhou, Zhejiang, China.

[†]These authors contributed equally.

[*]Correspondence: wulianfeng@westlake.edu.cn.

Contents

Supplementary Figure 1 Low dose of phenformin increased bacterial B12 accumulation over time.

Supplementary Figure 2 B12 level changes by phenformin treatment in tested bacterial strains with indicated genotypes.

Supplementary Figure 3 Scoring criteria of the primary and secondary screens for genes involved in Phen-induced bacterial B12 accumulation.

Supplementary Figure 4 Phenformin retarded the growth of all four tested *E. coli* strains.

Supplementary Figure 5 The growth inhibition effect of 5 mM phenformin on bacteria could be bacteriostatic.

Supplementary Figure 6 The deletion of *rcdA* did not affect phenformin accumulation in BW25113.

Supplementary Figure 7 Working model of metformin action in inducing bacterial B12 accumulation.

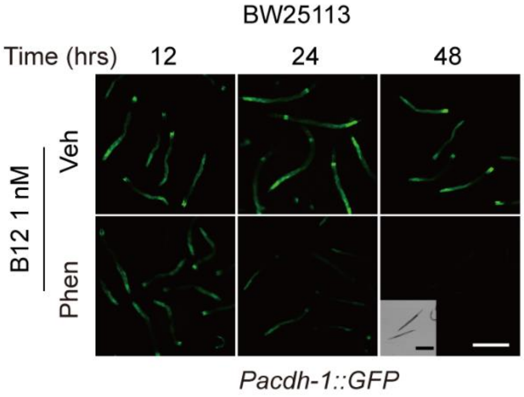

Supplementary Figure 1. Low dose of phenformin increased bacterial B12 accumulation over time. Representative images of *Pacdh-1::GFP* worms fed with BW25113 that were pretreated with B12 and/or 2 mM phenformin for indicated times. N = 3 independent experiments containing at least 30 worms per condition. Scale bar: 250 μm.

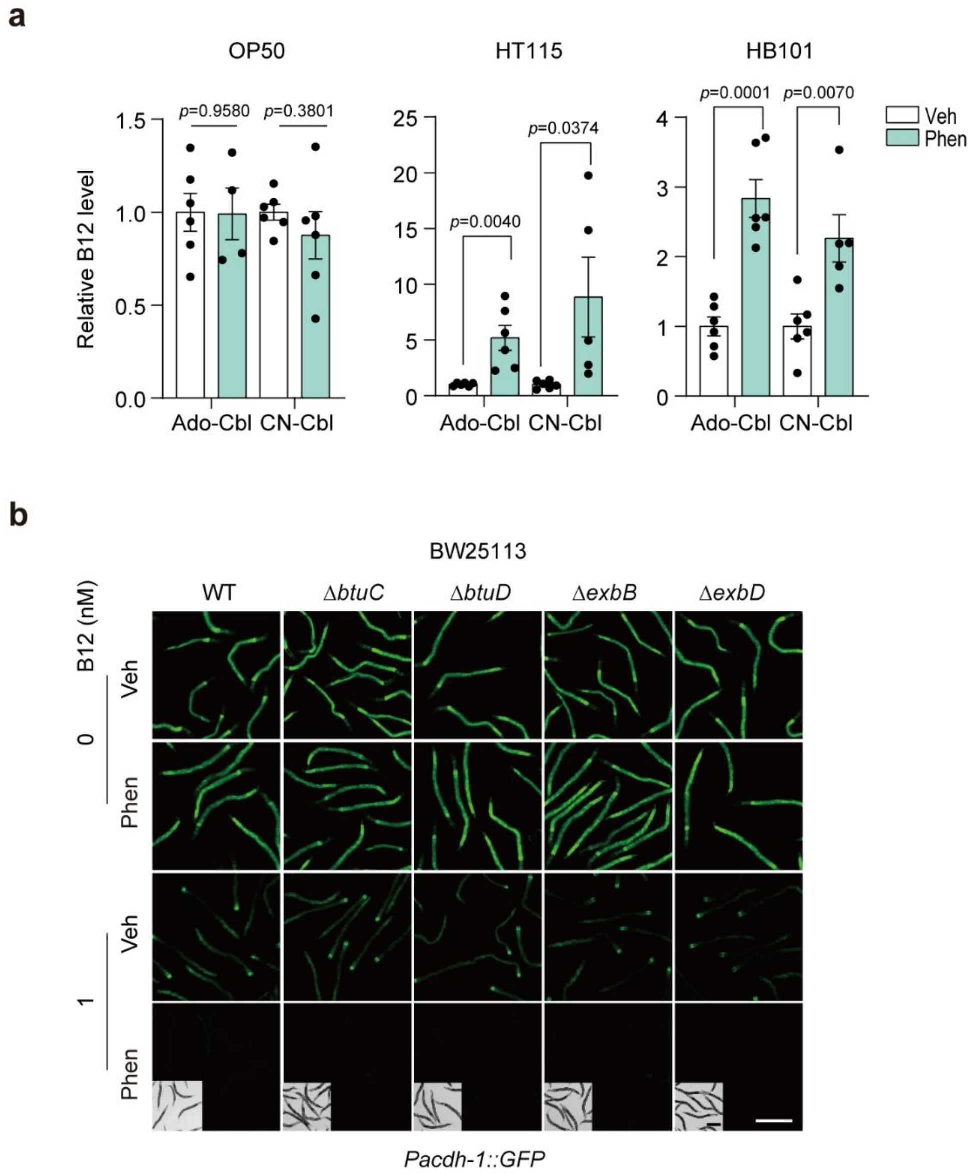

Supplementary Figure 2. B12 level changes by phenformin treatment in tested bacterial strains with indicated genotypes. a LC–MS/MS measurement of B12 levels in OP50, HT115 and HB101 treated with 4 mM phenformin. N =3 independent experiments with 2 single colonies each time. The statistical significance values were determined by multiple t tests. Error bars denoted the S.E.M. **b** Representative images of *Pacdh-1::GFP* worms fed with the B12 transporter mutant strains pretreated with B12 and/or 4 mM phenformin. N = 3 independent experiments containing at least 30 worms per condition. Scale bar: 250 μm.

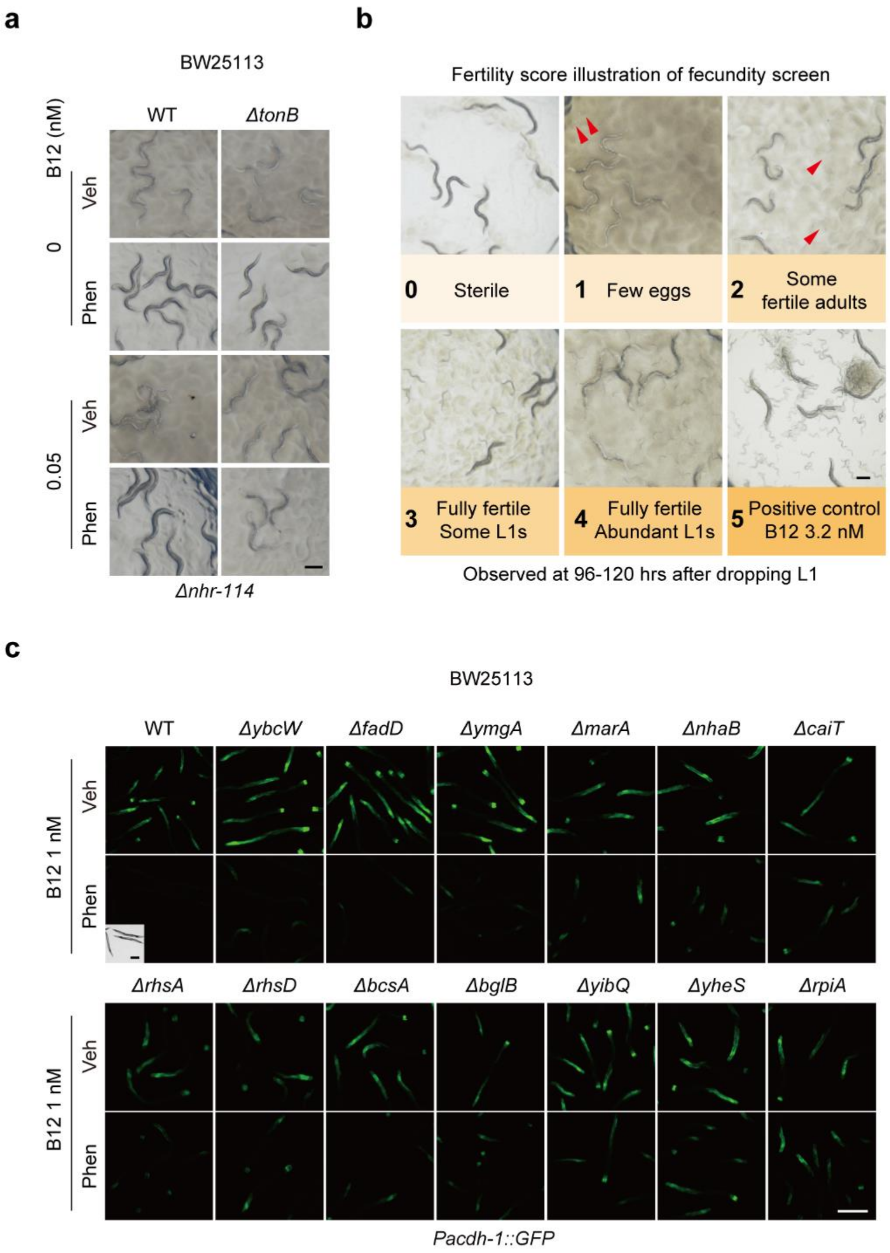

Supplementary Figure 3. Scoring criteria of the primary and secondary screens for genes involved in Phen-induced bacterial B12 accumulation. a Representative bright field images of *Δnhr-114* worms fed with WT and *ΔtonB* bacteria pretreated with 0.05 nM B12 and/or 2 mM phenformin. **b** Representative bright field images of *Δnhr-114* worms in the primary screen. Arbitrary scores 1-5 represented the overall fecundity outcomes of worms in tested wells. The red arrows indicated the eggs. **c** Representative

images of *Pacdh-1::GFP* worms fed with bacterial mutant candidates from the secondary screen. Candidates pretreated with B12 and/or 4 mM phenformin with GFP intensity scores greater than 1 were presented. N =3 independent experiments containing at least 30 worms per condition for panels (a-c). Scale bar: 250 μm.

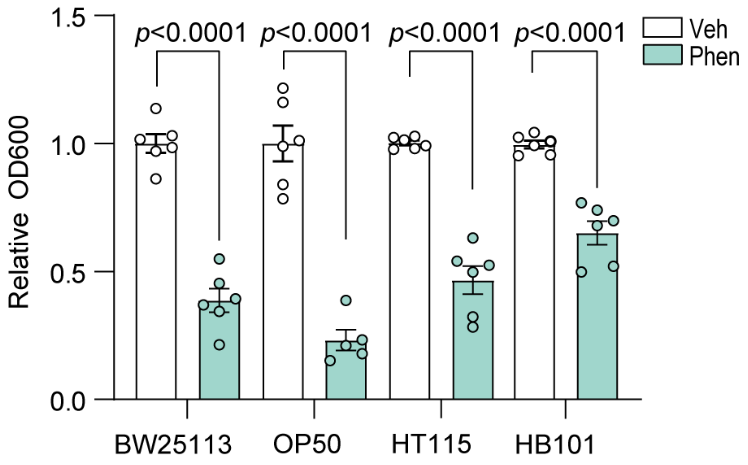

Supplementary Figure 4. Phenformin retarded the growth of all four tested *E. coli* strains. Relative OD600 values of BW25113, OP50, HT115, and HB101 treated with 4 mM phenformin compared to untreated ones. N = 3 independent experiments containing 6 replicates. The statistical significance values were determined by multiple t tests. Error bars denoted the S.E.M.

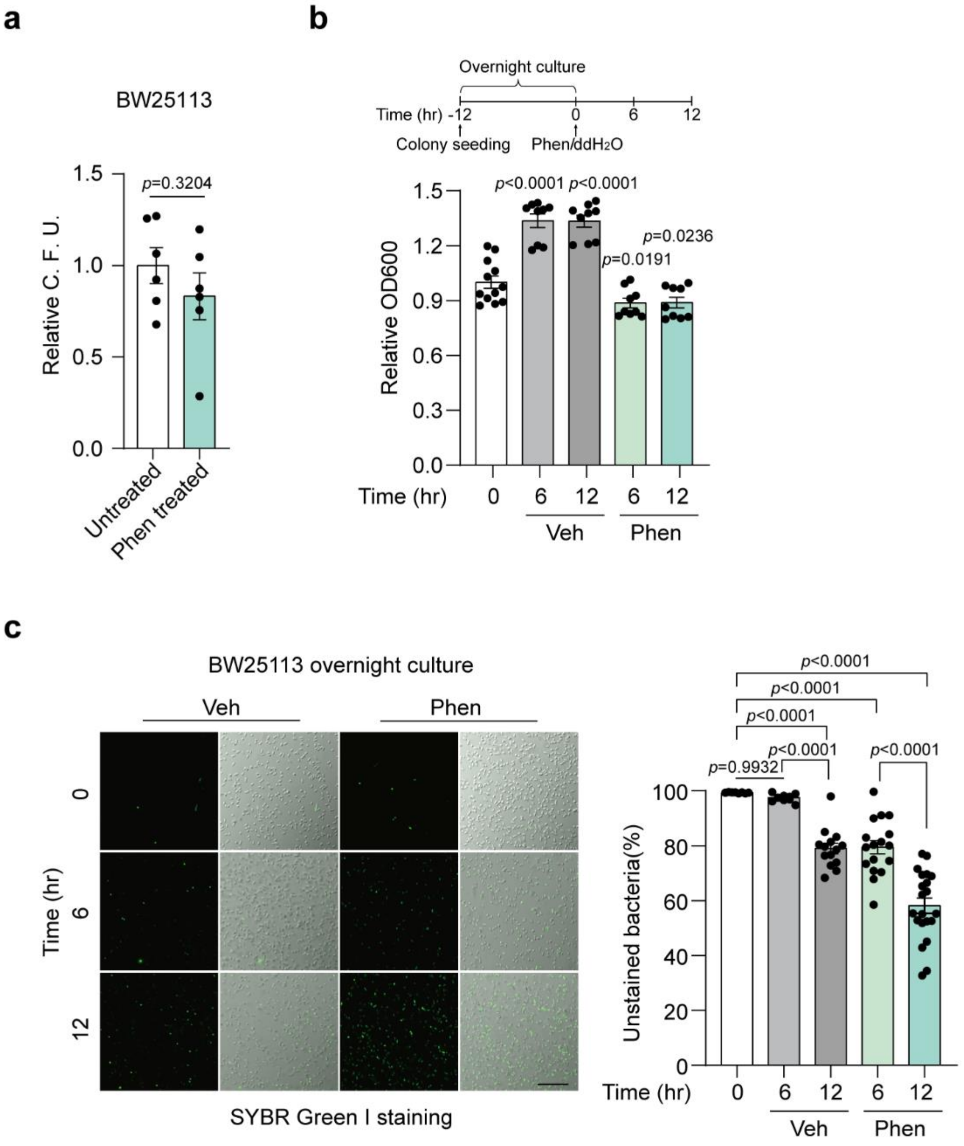

Supplementary Figure 5. The growth inhibition effect of 5 mM phenformin on bacteria could be bacteriostatic. a Relative C.F.U. of BW25113 before and after 5 mM phenformin treatment for 12 hours. N = 3 independent experiments containing 6 replicates. The statistical significance values were determined by unpaired t test. Error bars denoted the S.E.M. **b** Growth of overnight-cultured BW25113 treated with 5 mM phenformin or not for the indicated times. **c** SYBR green I staining of overnight-cultured BW25113 treated with 5 mM phenformin or not for the indicated times. Scale bar: 20 μm. N = 2 independent experiments containing 5-6 replicates. The statistical

significance values were determined by ordinary one-way ANOVA. Error bars denoted the S.E.M.

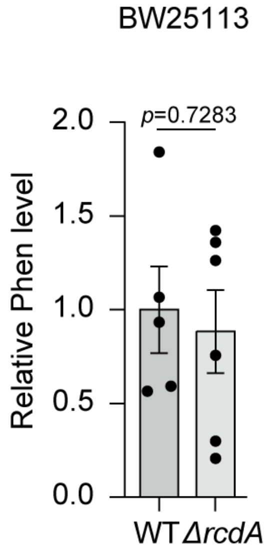

Supplementary Figure 6. The deletion of *rcdA* did not affect phenformin accumulation in BW25113. LC–MS/MS measurement of phenformin levels in WT and *ΔrcdA* treated with 4 mM phenformin. N = 2 independent experiments containing 6 replicates. The statistical significance value was determined by unpaired t test. Error bars denoted the S.E.M.

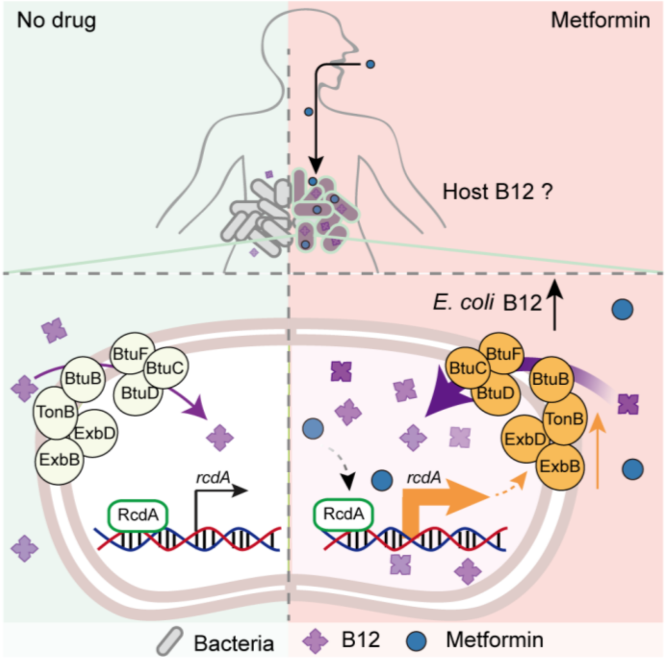

Supplementary Figure 7. Working model of metformin action in inducing bacterial B12 accumulation. Metformin treatment increases the expression of *rcdA,* which in turn elevates levels of B12 transporters. The enhanced capacity of intestinal bacterial B12 absorption may compete with the host for B12 resources and result in B12 deficiency in long-term metformin users.
