# Supplementary material for: The antidiabetic drug metformin aids bacteria in hijacking vitamin B12 from the environment through RcdA
Source: Commun Biol. 2023 Jan 24;6:96. doi: 10.1038/s42003-023-04475-0 (PMC9873799; doi:10.1038/s42003-023-04475-0)
Supplement: Supplementary file 3 — Description of Additional Supplementary Files [file 42003_2023_4475_MOESM3_ESM.pdf]

Notes of additional supplementary files

File name: Supplementary Data 1

Description: The outcomes of the primary screen

File name: Supplementary Data 2

Description: The information of composition for axenic culture medium

File name: Supplementary data 3

Description: The primers used in this study

File name: Supplementary data 4

Description: The source data for figures presented in the paper
